# MOF-Enabled Nanocellulose Composite Threads for Sustained Antibacterial Drug Delivery and Minimally Invasive Soft-Tissue Lifting

**DOI:** 10.3390/polym18101186

**Published:** 2026-05-12

**Authors:** Meng Sun, Meiyan Wu, Ping Wang, Bing Li, Guang Yu, Haishun Du, Tao Lou, Bin Li

**Affiliations:** 1 College of Chemistry and Chemical Engineering, Qingdao University, Qingdao 266071, China; s13127235826@163.com; 2 State Key Laboratory of Photoelectric Conversion and Utilization of Solar Energy, Qingdao New Energy Shandong Laboratory, System Integration Engineering Center, Qingdao Institute of Bioenergy and Bioprocess Technology, Chinese Academy of Sciences, Qingdao 266101, China; wumy@qibebt.ac.cn (M.W.); wangping@qibebt.ac.cn (P.W.); yuguang@qibebt.ac.cn (G.Y.); 3 Qingdao Hospital of Traditional Chinese Medicine (Municipal Hiser Hospital), Qingdao 266033, China; libing@qdu.edu.cn; 4 Department of Forestry, Michigan State University, East Lansing, MI 48824, USA; hdu@msu.edu

**Keywords:** carboxymethyl cellulose nanofibrils, sodium alginate, thread lifting, antibacterial property

## Abstract

Minimally invasive thread lifting has emerged as an effective strategy for soft tissue repositioning and facial rejuvenation; however, currently used absorbable threads generally lack intrinsic antimicrobial functionality, which may increase the risk of postoperative infection. Here, we report a biodegradable antibacterial lifting thread based on a nanocellulose/MOF composite system. The thread was fabricated via a green wet-spinning strategy using carboxymethylated cellulose nanofibrils (CCNF, prepared with cellulose derived from *Astragalus* residue) and sodium alginate (SA) as the structural matrix, while tetracycline hydrochloride-loaded ZIF-8 nanoparticles were incorporated to provide sustained antibacterial activity. The resulting antibacterial CCNF/SA thread (AB-CCNF/SA) exhibited a uniform morphology and a tensile strength of 80 MPa. The porous ZIF-8 carriers enabled efficient drug loading and controlled release, providing effective antibacterial activity against *Staphylococcus aureus* and *Escherichia coli*. Meanwhile, the composite threads showed favorable biodegradability, with approximately 45% degradation within 56 days, together with excellent cytocompatibility as demonstrated by fibroblast viability above 90%. In vivo studies further revealed inflammatory responses comparable to those of commercial collagen threads, confirming the good biocompatibility of the system. Overall, this work establishes a strategy for integrating nanocellulose structural materials with MOF-enabled antibacterial drug delivery, providing a multifunctional platform that combines mechanical support, biodegradability, and sustained antibacterial activity for minimally invasive soft tissue lifting and related biomedical implant applications.

## 1. Introduction

Facial skin is continuously exposed to external stressors such as UV radiation, pollutants, chemicals, and environmental toxins, which can progressively impair skin function over time. These changes are commonly characterized by reduced skin elasticity and facial volume, ultimately leading to wrinkles, dryness, weakened barrier function, and thinning of the epidermis [1,2,3,4]. To address these age-related changes and restore facial contours, minimally invasive aesthetic procedures have been increasingly developed in recent years [5,6]. Absorbable lifting threads are widely used in minimally invasive aesthetic procedures for soft tissue repositioning and facial rejuvenation. After implantation beneath the skin, these threads provide immediate mechanical support while gradually degrading and stimulating collagen deposition in surrounding tissues [7]. Compared with traditional surgical lifting procedures, thread lifting offers several advantages, including minimal trauma, shorter recovery time, and reduced surgical risks [8]. As a result, it has become an increasingly popular technique in cosmetic and reconstructive medicine. Currently used lifting threads are mainly composed of biodegradable polymers such as collagen, polydioxanone (PDO), and polylactic acid (PLA) [9]. These materials provide effective mechanical lifting and gradually degrade in vivo, thereby avoiding the need for surgical removal [10]. However, most clinically used lifting threads lack intrinsic antibacterial activity. Once implanted beneath the skin, the materials may be exposed to microbial contamination during the surgical procedure or post-operative healing process, which can increase the risk of infection and local inflammation. Therefore, developing lifting threads with integrated antimicrobial functionality threads is highly desirable for improving clinical safety.

Cellulose is a natural polymer composed of β-(1,4)-D-glucose units and is widely recognized as a renewable, biocompatible, environmentally friendly material with inherent biodegradability [11,12]. It is worth noting that the purity of cellulose significantly affects the preparation efficiency and final product performance of nanocellulose. Raw materials with high cellulose content (>80%) and low hemicellulose and lignin content (<10%) are more conducive to obtain nanocellulose with high aspect ratio and low defects [13,14,15]. However, the human body lacks cellulase enzymes, making conventional cellulose fibers difficult to enzymatically degrade after implantation [16]. In contrast, nanocellulose exhibits a much higher surface area and smaller dimensions, which may facilitate oxidative degradation and cellular clearance in biological environments [17]. Owning to its unique structural characteristics, as well as high specific strength and modulus and good biocompatibility, nanocellulose has attracted considerable interest in biomedical applications [18,19,20]. For example, Wu et al. prepared anionic cellulose nanofibril (ACNF)/cationic guar gum (CGG) composite threads via interfacial polyelectrolyte complex (IPC) spinning, which showed favorable wound-healing performance in both in vitro and in vivo experiments [21]. However, the IPC process often suffers from low efficiency, significant batch-to-batch variability, and the inherent difficulty of spinning pure nanocellulose, which limits its scalability and industrial application [22]. To overcome these limitations, incorporating sodium alginate (SA) and employing wet-spinning technology provides an effective strategy for fabricating nanocellulose-based threads. SA can form a homogeneous and stable spinning dope with nanocellulose, enabling continuous fiber formation while improving processability. Moreover, SA possesses excellent water-retention and moisturizing properties, which help maintain a moist microenvironment at wound sites and promote collagen deposition and angiogenesis [23]. For instance, Liu et al. successfully fabricated SA/cellulose nanocrystal (SA/CNC) threads, where the synergistic interaction between SA and CNC significantly enhanced both tensile strength and toughness [24]. Therefore, the nanocellulose/SA system, which is cost-effective and fully derived from renewable biopolymers, represents a promising candidate for biodegradable biomedical threads. Nevertheless, these natural components inherently lack antimicrobial functionality, which may increase the risk of bacterial colonization after implantation. Therefore, developing lifting threads with long-lasting and controllable antibacterial activity is essential for improving their safety and clinical performance.

To endow lifting threads with antibacterial functionality, incorporating antibacterial agents into the thread matrix represents an effective strategy. Metal–organic frameworks (MOFs), particularly ZIF-8, have attracted considerable interest as drug delivery carriers due to their porous structure, high loading capacity, controllable release behavior, and readily functionalized surface [25]. These characteristics enable ZIF-8 to efficiently encapsulates various therapeutic agents, including tetracycline hydrochloride (TH) [26], curcumin (Cur) [27], and doxorubicin (DOX) [28]. In addition, ZIF-8-based systems have demonstrated satisfactory antibacterial activity against *Staphylococcus aureus* (*S. aureus*) and *Escherichia coli* (*E. coli*) [29]. TH, a commonly used broad-spectrum antibiotic for treating numerous bacterial infections [30], was selected as the antibacterial drug in this study.

Herein, a biodegradable antibacterial lifting thread based on carboxymethyl cellulose nanofibrils (prepared with *Astragalus* residue) and sodium alginate (AB-CCNF/SA) was developed via a green wet-spinning method. Tetracycline-loaded ZIF-8 nanoparticles were incorporated to provide sustained antibacterial activity. The morphology, mechanical properties, degradation behavior, antibacterial performance, and biocompatibility of the threads were systematically evaluated. In addition, the in vivo tissue response was investigated using a rat subcutaneous implantation model. The results demonstrate that the developed AB-CCNF/SA threads combine mechanical support, biodegradability, and long-term antibacterial functionality, highlighting their potential for minimally invasive soft tissue lifting applications.

## 2. Materials and Methods

### 2.1. Material

Carboxymethyl cellulose nanofibrils (CCNF) with a low degree of substitution (DS) of 0.127 and a carboxyl content of 1.6 mmol/g were prepared from *Astragalus* fiber through an etherification reaction followed by high-pressure homogenization, preserving the native cellulose crystallinity and abundant hydroxyl groups for subsequent crosslinking. The detailed preparation procedure is provided in the Supporting Information document [31,32,33]. Calcium chloride (CaCl_2_), 2-methylimidazole (2-MIM), zinc nitrate hexahydrate (Zn(NO_3_)2·6H_2_O), methanol, and sodium chloride (NaCl) were purchased from Sinopharm Chemical Reagent Co., Ltd. (Shanghai, China). Sodium alginate (SA) and glutaraldehyde (GA) were purchased from Shanghai Aladdin Bio-Chem Technology Co., Ltd. (Shanghai, China). Tetracycline hydrochloride (TH) was purchased from Shanghai Maclin Bio-Chem Technology Co., Ltd. (Shanghai, China). Phosphate-buffered saline (PBS, pH 7.4) was obtained from Beijing Lanjieke Technology Co., Ltd. (Beijing, China). Commercial collagen lifting threads were supplied by Shandong Boda Medical Supplies Co., Ltd. (Heze, China). All chemical reagents were used as received without further purification.

### 2.2. Preparation of AB-CCNF/SA Threads

ZIF-8 nanoparticles and TH-loaded ZIF-8 were first prepared according to the procedure described in the Supporting Information [34,35,36]. CCNF and SA were then dispersed in deionized water at a mass ratio of 1:1 with a total solid content of 1.3 wt.%. The mixture was magnetically stirred for 20 min to obtain a homogeneous spinning dope. TH powder (6.0 wt.% relative to the total mass of CCNF/SA solids) was subsequently added, and the mixture was further stirred at room temperature for 20 min. The spinning solution was extruded into a 5 wt.% CaCl_2_ coagulation bath using a syringe pump equipped with a stainless-steel needle (inner diameter: 0.51 mm; length: 25 mm) at an extrusion rate of 0.5 mm/min [37]. The formed filaments were thoroughly washed with deionized water and air-dried at 25 °C under 40% relative humidity (RH). The dried threads were subsequently immersed in a GA crosslinking solution for 1 h, followed by thermal treatment at 130 °C for 0.5 h to obtain antibacterial CCNF/SA composite threads (AB-CCNF/SA).

### 2.3. Characterization

The morphology, structure, and elemental composition of the raw materials and threads were characterized using a polarizing stage microscope (Guangzhou Mingmei Optoelectronic Technology, MP41, Guangzhou, China) and a scanning electron microscope (SEM, HITACHI, S-4800, Hitachi, Japan). Prior to SEM observation, all samples were mounted on conductive adhesive and sputter-coated with a thin layer of gold for 60 s to enhance electrical conductivity. X-ray diffraction (XRD) patterns were recorded using a diffractometer (Rigaku, SmartLab 9KW, Takatsuki, Japan) equipped with a Cu Kα radiation. The instrument was operated at 30 kV and 30 mA with a scanning range of 5–60° (2θ). Attenuated Total internal Reflectance Fourier Transform Infrared (ATR-FTIR) spectra were obtained using a spectrometer (Thermo Fisher Scientific, Nicolet iS50, Waltham, USA) in the wavenumber range of 4000–400 cm^−1^.

The specific surface area and porosity of ZIF-8 and drug-loaded ZIF-8 were analyzed by nitrogen adsorption–desorption measurements using a BET surface area analyzer (Belsorp Max X, Osaka, Japan). Prior to measurement, samples were degassed under vacuum at 150 °C for 12 h. The adsorption–desorption isotherms were collected at −196 °C using N_2_ as the adsorbate over a relative pressure (P/P_0_) range of 0–1 with approximately 50 mg of sample for each analysis [27]. Thermogravimetric analysis was conducted using a thermal analyzer (NETZSCH, STA 449F5 Jupiter, Selb, Germany) in the temperature range of 50–600 °C at a heating rate of 10 °C/min under a nitrogen atmosphere (25 mL/min). Tensile properties of the threads were measured using a universal testing machine (MTS Systems, CMT 6503, Ningbo, China). The samples were cut into 20 mm lengths and tested at 25 °C and 50% RH with a load cell of 100 N and a crosshead speed of 1 mm/min. Each sample was tested at least three times, and the standard deviation was maintained below 5%.

### 2.4. In Vitro Experiments

AB-CCNF/SA threads (0.03 g) were immersed in 10 mL of PBS (pH 7.4) and physiological saline solution at 37 °C. At predetermined time intervals, 3 mL aliquots of the release medium were withdrawn and replaced with an equal volume of fresh medium to maintain sink conditions. The TH concentration in the collected samples was quantified using UV-visible spectrophotometry (Aoe Instruments, UV-1800, Shanghai, China) at 360 nm. The cumulative drug release (%) was calculated according to Equation (1):

*Cumulative release* (%) = (1 × Σ *C_i_*_−1_ + 10*C_i_*) × 10^−3^/*m* × 100% (1)

where *C_i_* represents the concentration of TH in the release medium at time *i* (mg/L), and *m* is the total mass of TH incorporated in the thread (mg).

The in vitro biodegradation behavior of AB-CCNF/SA and commercial collagen threads was evaluated by measuring the loss of tensile strength during degradation. The threads were immersed in physiological saline for a predetermined period, gently rinsed with deionized water, and blotted with filter paper to remove excess moisture. The residual tensile strength was then measured to calculate the strength loss. Detailed preparation procedures [38,39,40] for other experimental steps are available in the Supporting Information.

### 2.5. In Vivo Experiments

All animal procedures were conducted in a specific pathogen-free (SPF) facility and approved by the Ethics Committee of Qingdao Hospital of Traditional Chinese Medicine (Approval No. 2024HC01LS24).

Subcutaneous implantation in mice was performed to evaluate thread biocompatibility and tissue inflammatory response of the threads. Six-week-old male and female SKH-1 hairless mice (Orient Bio Inc., Seoul, South Korea) weighing 16–26 g were acclimatized for one week under controlled conditions (24 °C, 55% RH, 12 h light/dark cycle) with free access to food and water. The animals were randomly divided into treatment groups (*n* = 5). Under isoflurane anesthesia, the dorsal skin was disinfected with ethanol solution. Subsequently, two threads were implanted subcutaneously in each mouse at positions approximately 1 cm from the spine. Hemostasis was achieved by gentle compression, followed by post-operative disinfection. After 21 days of implantation, the animals were euthanized under anesthesia. Dorsal skin tissues were excised, fixed in 4% (*v*/*v*) paraformaldehyde, and sectioned into 20 μm-thick frozen sections. Hematoxylin and eosin (H&E) staining was performed to evaluate epidermal and dermal morphology. In addition, interleukin-6 (IL-6) expression was analyzed by immunohistochemistry to assess inflammatory responses and tissue remodeling, including vascularization, macrophage infiltration, and hair follicle regeneration. Histological images were captured using a laser scanning microscope (Guangzhou Mingmei Optoelectronic Technology, MI52-N, Guangzhou, China) and analyzed with KFSlideOS software (version 1.0.8, KFBIO, Yuyao City, China), using normal skin tissue as the control.

### 2.6. Statistical Analysis

All data were presented as mean ± standard deviation (SD) unless otherwise specified. Statistical analysis of optical density values was performed using SPSS 16.0 software with one-way analysis of variance (ANOVA). Differences were considered statistically significant when *p* < 0.05. Graphs were generated using Origin 2018 software.

## 3. Results and Discussion

### 3.1. Fabrication of AB-CCNF/SA Threads

**Figure 1:**
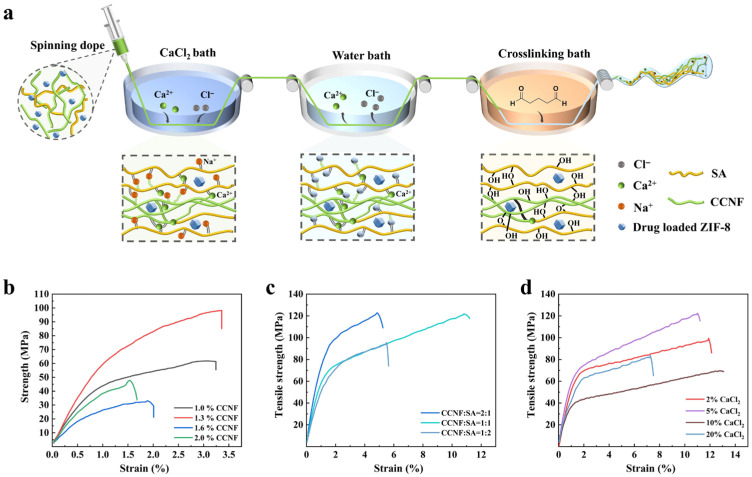
Fabrication and optimization of AB-CCNF/SA threads. (**a**) Schematic illustration of the threads fabrication process, (**b**) Tensile strength of threads prepared with different solid contents of CCNF spinning solution, (**c**) Tensile strength of threads with varying CCNF/SA ratios, (**d**) Tensile strength of threads prepared using coagulation baths with different CaCl_2_ concentrations.

As illustrated in Figure 1a, AB-CCNF/SA threads were fabricated by incorporating TH-loaded ZIF-8 nanoparticles into a CCNF/SA spinning solution, followed by wet spinning and subsequent crosslinking. Briefly, CCNF and SA were first co-dispersed under magnetic stirring to form a homogeneous suspension. The ZIF-8/TH powder was then added and further homogenized using a high-pressure homogenizer to obtain a uniform pale-yellow viscous spinning dope.

The effect of CCNF solid content on the tensile properties of the spun threads was first investigated. As shown in Figure 1b, when the solid content of the CCNF dispersion increased from 1.0% to 2.0%, the tensile strength of the threads initially increased and then decreased, reaching a maximum value of 98 MPa at 1.3%. At low solid contents, the resulting threads were relative thin and prone to structural defects, whereas excessively high solid contents resulted in highly viscous spinning dopes (Figure S2), leading to structural inhomogeneity during thread formation. Both conditions negatively affected the mechanical strength of the threads. However, the pristine CCNF threads exhibited limited flexibility, with a fracture elongation of only 3.4% (Figure 1b), which restricts their practical applications. To improve the mechanical performance, composite threads were prepared by incorporating sodium alginate (SA) into the CCNF matrix at the optimal solid content of 1.3%. As shown in Figure 1c, the introduction of SA significantly improved the elongation at break of the threads. In particular, at a CCNF/SA mass ratio of 1:1, the composite threads achieved an elongation at break of 11% together with an enhanced tensile strength of 122 MPa. Meanwhile, the viscosity of the CCNF/SA composite spinning solution remained comparable to that of the pure CCNF suspension (Figure S2), which is beneficial for stable thread formation.

The influence of coagulation bath concentration on the mechanical properties of the CCNF/SA (1:1) threads was further investigated using CaCl_2_ solutions ranging from 2% to 20% (Figure 1d). The gelation mechanism is based on ionotropic crosslinking, wherein Ca^2+^ ions rapidly crosslink SA chains through the formation of egg-box complexes with guluronic acid blocks, followed by gradual coordination with the surface carboxyl groups (-COOH) of both CCNF and SA [11]. The Ca^2+^ mediated coordination leads to the formation of a denser crosslinked network, thereby enhancing the mechanical strength and structural stability of the threads. At low CaCl_2_ concentrations (2%), insufficient crosslinking and gelation resulted in loosely structured fibers. In contrast, excessively high Ca^2+^ concentrations (>10%) induced over-gelation and rapid viscosity increases, which hindered molecular rearrangement and led to brittle fracture behavior with reduced tensile properties. Therefore, an optimal mechanical performance was achieved at a CaCl_2_ concentration of 5%.

To further introduce antibacterial functionality and improve water resistance, TH-loaded ZIF nanoparticles and a chemical crosslinker were incorporated into the CCNF/SA threads. After optimization, the composite threads containing 6 wt% TH (relative to the dry mass of CCNF/SA) exhibited favorable antibacterial properties. Subsequently, crosslinking was carried out in a 2% GA solution [41]. The aldehyde groups in the GA molecules could react with the hydroxyl groups of SA and CCNF to form acetal bonds, constructing a covalent crosslinking network. Therefore, GA acted as a molecular bridge connecting SA and CCNF to form an interpenetrating network structure [42], further improving the structural stability of the fiber (Figures S3 and S4).

Based on the above results, the optimal spinning conditions were determined to be a CCNF/SA solid content of 1.3% with a mass ratio of 1:1, a 5% CaCl_2_ coagulation bath, 6 wt% TH loading, and 2% GA crosslinker. Threads prepared under these conditions exhibited the best mechanical performance and antibacterial properties and were therefore used in all subsequent experiments.

### 3.2. Drug Loading of ZIF-8

**Figure 2:**
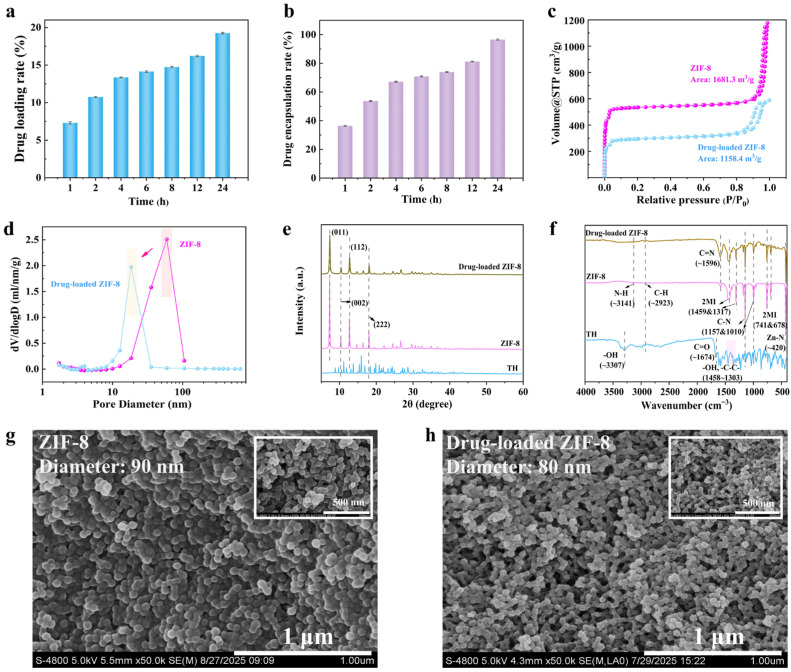
Characterization of ZIF-8 before and after drug loading. (**a**) Drug loading capacity and (**b**) encapsulation efficiency of ZIF-8 as the function of incubation time for drug loading, (**c**) Nitrogen adsorption–desorption isotherms, (**d**) BJH pore size distribution, (**e**) XRD patterns, (**f**) FTIR spectra and (**g**,**h**) SEM images of ZIF-8 and TH-loaded ZIF-8.

Based on the calibration curve (Figure S5), the loading behavior of TH in methanol was evaluated at different incubation time. As shown in Figure 2a,b, the drug loading capacity gradually increased with incubation time and reached a maximum value of 19.3% at 24 h, with an encapsulation efficiency of 97.1%. The high drug loading capacity can be attributed to several factors: (i) the inherent high specific surface area of ZIF-8 provides abundant adsorption sites for drug molecules [43]; (ii) the enhanced pore accessibility of ZIF-8 in methanol facilitates the diffusion and retention of TH molecules; and (iii) in the methanol system, the weakened hydration effect increases the exposure of functional groups, thereby strengthening intermolecular interactions, including hydrogen bonding (TH-OH/-NH with imidazole N), π-π interactions (TH aromatic ring with imidazole ring), and hydrophobic interactions between TH hydrophobic backbone and the inner surface of ZIF-8) [44].

The BET specific surface area of pristine ZIF-8 was 1681.3 m^2^/g, indicating the formation of a well-defined crystalline porous framework, which is comparable to the reported value of 1753.4 m^2^/g [45]. After TH loading, the surface area decreased to 1158.4 m^2^/g, suggesting partial occupation of the pores by TH molecules. As shown in Figure 2c, the nitrogen adsorption–desorption isotherms of both ZIF-8 and TH-loaded ZIF-8 exhibit typical type I isotherms, characterized by rapid nitrogen uptake at low relative pressure (P/P_0_ < 0.1), indicating a predominantly microporous structure. However, TH-loaded ZIF-8 showed a lower adsorption capacity and a more pronounced hysteresis loop, suggesting partial pore blockage and structural modification. The BJH pore size distribution analysis further revealed distinct differences between the two samples (Figure 2d). Pristine ZIF-8 displayed a dominant mesopore peak at approximately 50 nm (2.5 mL/nm/g), whereas TH-loaded ZIF-8 exhibited a shifted primary peak at around 20 nm with a reduced intensity (2.0 mL/nm/g) and a narrower pore size distribution (5–40 nm). This shift toward smaller pore sizes suggests partial pore filling or framework contraction induced by TH loading.

The crystalline structure of ZIF-8 before and after drug loading was further examined by XRD (Figure 2e). Both samples displayed characteristic diffraction peaks at 2θ = 7.31°, 10.32°, 12.71°, and 18.04°, corresponding to the (011), (002), (112), and (222) crystal planes of ZIF-8, respectively, confirming that the crystalline framework remained intact after drug loading. However, a slight decrease in peak intensity was observed for TH-loaded ZIF-8, and the crystallinity decreased by about 57% (calculated based on the previous report [46]. The FTIR spectra (Figure 2f) further verified the structural characteristics of the samples. The absorption band at 3141 cm^−1^ corresponds to N-H stretching vibration, while the peaks near 2920 and 1596 cm^−1^ are attributed to the stretching vibrations of aromatic C-H and C=N bonds, respectively. The peaks at approximately 1157 and 1010 cm^−1^ correspond to C-N stretching vibrations of the imidazole ring. In addition, the bands in the range of 1459–1317 cm^−1^ and 741–678 cm^−1^ correspond to in-plane bending and out-of-plane vibration modes of the imidazole ring, while the Zn-N stretching vibration appears at 420 cm^−1^ [47]. After drug loading, the intensity of these characteristic peaks decreased to varying degrees. Notably, the absence of independent -OH and C=O peaks associated with TH suggests that the drug molecules were encapsulated within the ZIF-8 framework rather than simply adsorbed on the surface.

The SEM images (Figure 2g,h) further reveal slight particle aggregation after drug loading, which may result from surface-adsorbed TH molecules or minor framework perturbation. Collectively, these structural and morphological analyses confirm the successful encapsulation of TH within ZIF-8 framework. The high drug loading capacity provides a solid basis for the sustained antibacterial activity of the resulting AB-CCNF/SA threads.

### 3.3. Characterization and Drug Release of the Threads

**Figure 3:**
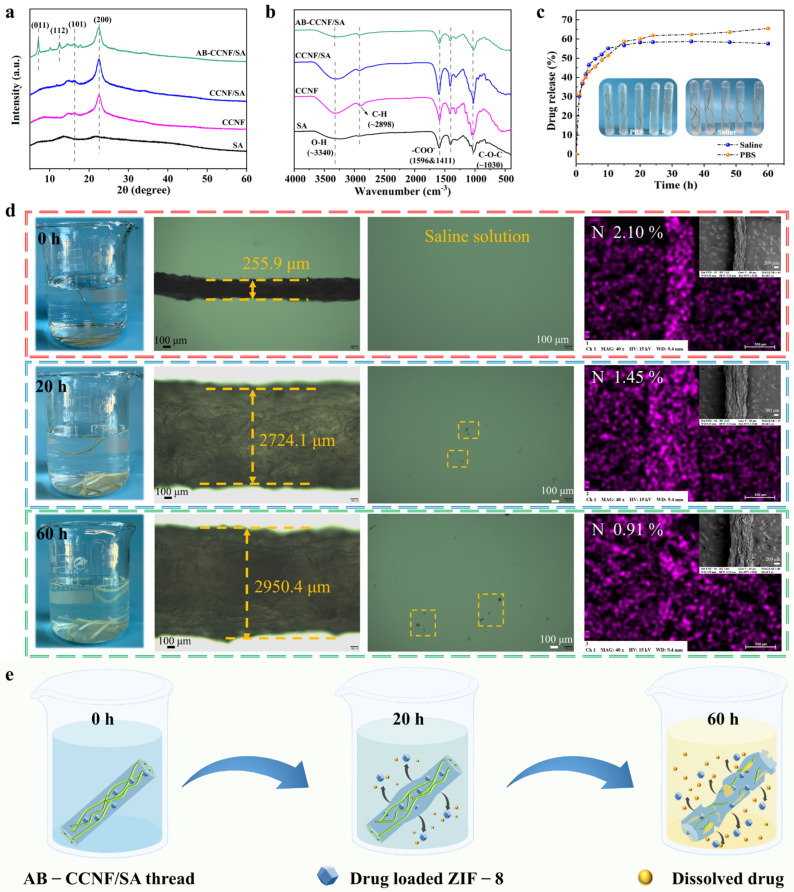
Characterization of AB-CCNF/SA threads. (**a**) XRD patterns and (**b**) FTIR spectra of the raw materials and threads, (**c**) Drug release profiles, (**d**) Digital images and SEM-EDS mappings of the threads after immersion in saline (with the indication of corresponding N content), (**e**) Schematic illustration of the biodegradation mechanism of AB-CCNF/SA thread.

Drug-loaded ZIF-8 nanoparticles were integrated into the CCNF/SA spinning solution to fabricate AB-CCNF/SA composite threads. The crystalline structure of the resulting threads was first examined by XRD. As shown in Figure 3a, CCNF exhibited two characteristic diffraction peaks at 2θ = 16.4°, 22.6°, corresponding to the (101) and (200) crystallographic planes of cellulose Iβ [48]. These peaks were preserved in the AB-CCNF/SA thread. In addition, new diffraction peaks appeared at 7.3° and 12.7°, corresponding to the (011) and (112) planes of the drug-loaded ZIF-8 [34]. The presence of these characteristic diffraction peaks confirms the successful incorporation of drug-loaded ZIF-8 nanoparticles into the thread matrix. The FTIR spectra further verified the structural composition of the materials (Figure 3b). The characteristic absorption bands of CCNF, CCNF/SA, and AB-CCNF/SA appeared at similar wavenumbers, indicating that the fundamental chemical structure of the cellulose network remained unchanged after composite formation. However, the AB-CCNF/SA thread exhibited relatively reduced absorption intensities for the carboxyl (-COO^−^) and ether (C-O-C) groups compared with the CCNF/SA control. This change suggests interactions between the polymer matrix and ZIF-8 nanoparticles, further confirming the successful integration of drug-loaded ZIF-8 into the CCNF/SA matrix.

After confirming the successful incorporation of drug-loaded ZIF-8, the drug release behavior of the threads was evaluated under simulated physiological conditions. PBS and saline were selected as release media to mimic the human physiological environment. The cumulative release of TH was calculated based on the calibration curves shown in Figure S6a,b. As illustrated in Figure 3c, the drug release profile exhibited a typical biphasic pattern, consisting of an initial rapid release followed by a sustained release stage. The cumulative release reached approximately 60% within 20 h without a pronounced burst release, and this performance is better in comparison to the ZIF-8@hyaluronic acid hydrogel system (more than 80% TH released rapidly within 3 h, and then a gradual release until 8 h, at pH 5.5 in PBS solution) [49] and the hyaluronic acid/alginate scaffold system (burst release in the first 1 h (50%) and the maximum TH release (85%) was achieved within 20 h, at pH 7.4 in PBS solution) [50]. This result indicates that the microporous structure of ZIF-8 effectively regulates the sustained release of TH.

To further elucidate the release mechanism, the drug release data were fitted to several classical kinetic models (Figure S6c–f). Among them, the Korsmeyer-Peppas (K-P) model provided the best fitting results, with correlation coefficients (R^2^) higher than 0.96 for all samples (Table S1). The release exponent (*n*) values were consistently below 0.5, indicating that the drug release follows a Fickian diffusion-controlled mechanism [51]. These results suggest that the release of TH is primarily governed by diffusion through the ZIF-8 framework, while the surrounding polymer network acts as a structural diffusion barrier.

Meanwhile, digital and microscopic observations revealed that the AB-CCNF/SA threads gradually swelled during immersion in saline. The thread diameter increased from 255.9 μm to 2950.4 μm, accompanied by progressive surface erosion and partial material shedding (Figure 3d). The GA crosslinking treatment helped maintain the structural integrity of the threads during this process, preventing rapid disintegration. SEM-EDS mapping further indicated that nitrogen signals, corresponding to TH molecules, were initially uniformly distributed throughout the thread matrix. After 60 h of degradation, the nitrogen signals became discontinuous and sparsely distributed (Figure 3d), indicating gradual drug release during the degradation process. These observations suggest a degradation-assisted release mechanism, in which the AB-CCNF/SA threads first undergo hydration and swelling, followed by gradual structural erosion, ultimately enabling the sustained release of encapsulated TH molecules (Figure 3e).

### 3.4. In Vitro Application Performances

Implantable medical threads require appropriate biodegradability, biocompatibility, and minimal contamination to reduce tissue damage and foreign body reactions. Collagen threads are widely used in facial lifting procedures because of their excellent biocompatibility and established clinical performance. Therefore, commercial collagen threads were selected as a reference to evaluate the application potential of AB-CCNF/SA threads.

**Figure 4:**
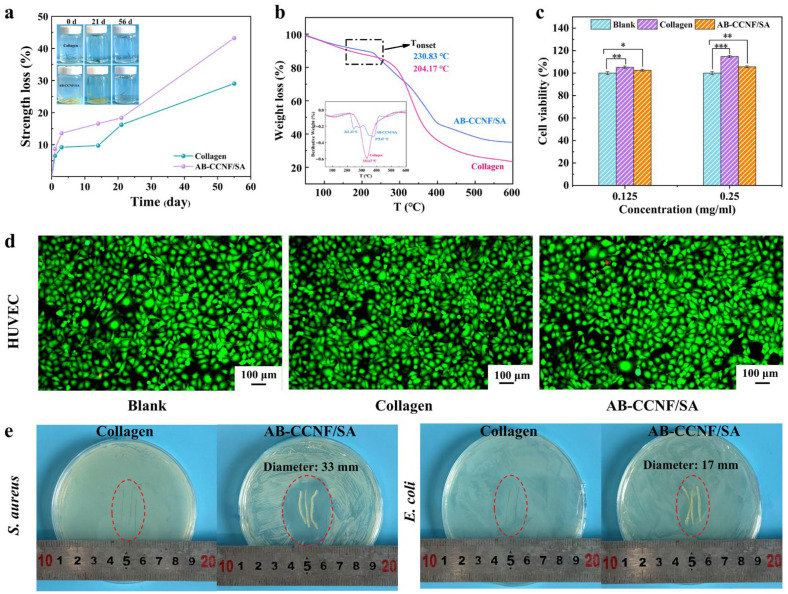
Degradation, cytocompatibility, and antibacterial ability of thread samples. (**a**) Strength loss of AB-CCNF/SA and collagen threads during degradation in saline, (**b**) Thermogravimetric analysis curves of AB-CCNF/SA and collagen threads, (**c**) cell viability results of AB-CCNF/SA and collagen threads, * *p* < 0.05, ** *p* < 0.01,*** *p* < 0.001, *p* represents the probability (significance) that the observed differences arise from random error, *n* = 5, (**d**) LIVE/DEAD cell viability assay results, green fluorescence indicates viable cells, while red fluorescence indicates dead cells, (**e**) Antibacterial effects of AB-CCNF/SA and collagen threads against *E. coli* and *S. aureus*.

The solubility of medical threads is typically assessed by monitoring the loss of tensile strength during degradation. As shown in Figure 4a, AB-CCNF/SA threads immersed in saline exhibited similar solubility behavior to collagen sutures: approximately 13.6% of initial tensile strength was lost in the first 3 days, approximately 16.6% in the first 14 days, and approximately 18.4% by day 21 (81.6% retained, 35.6 MPa). Although the solubility rate of AB-CCNF/SA threads was slightly faster than that of collagen threads (16.2% loss at day 21, 83.8% retained, 32.6 MPa), their high initial strength (43.6 MPa vs. 39.0 MPa) meant that their strength during the critical postoperative support period (within 21 days) remained superior to that of collagen threads, sufficient to meet the mechanical requirements of skin soft tissue repositioning and early collagen deposition. By day 55, 43.3% of the AB-CCNF/SA threads had been lost (56.7% retained, 24.7 MPa), indicating that the tissue had begun to heal and the reliance on artificial support had significantly decreased. This characteristic of “early support, mid-term stability, and late-term absorption” dynamically matches the biomechanical requirements after skin and soft tissue lifting surgery—support during the critical period ensures stable lifting effects, while late-term degradation avoids long-term foreign body retention and the risk of secondary surgery, thus improving patient safety while ensuring clinical efficacy.

Thermogravimetric analysis (TGA) was further performed to assess the thermal stability of the threads (Figure 4b). Both samples exhibited an initial weight loss in the temperature range of 50–200 °C, which can be attributed to the evaporation of physically adsorbed moisture. Collagen threads showed the onset of rapid thermal decomposition at approximately 204 °C, whereas AB-CCNF/SA threads displayed a more gradual degradation process and produced a higher char residue at elevated temperatures. The derivative thermogravimetric (DTG) curves further revealed that the maximum decomposition temperature of AB-CCNF/SA threads was higher than that of collagen threads, improved thermal stability of the composite system. TGA has been widely used to characterize the thermal degradation behavior of absorbable sutures because the decomposition curves can reflect the inherent structural stability and degradation kinetics of biopolymer sutures under thermal stress. Furthermore, TGA studies of albumin-based absorbable sutures also showed that thermal stability is directly related to the material’s ability to withstand sterilization treatment [52]. For example, collagen-based absorbable sutures typically exhibit an initial degradation temperature of approximately 200 °C, consistent with the collagen sutures evaluated in this study [53]. This enhanced thermal stability suggests that the threads can tolerate standard sterilization processes (e.g., autoclaving at 121 °C) without structural damage, thereby ensuring surgical safety and processing convenience.

Cytocompatibility is another key parameter for evaluating the biological safety of implant materials [54]. In this study, the cytocompatibility of thread extracts was assessed using human umbilical vein endothelial cells (HUVEC). As shown in Figure 4c,d, HUVEC viability remained close to 100% after 24 h incubation with AB-CCNF/SA thread extracts at concentrations of 0.15 and 0.25 mg/mL. Notably, the cell viability was slightly higher than that of the blank control group, suggesting that the extracts did not inhibit cell growth and may even support cell proliferation. These results indicate that AB-CCNF/SA threads exhibit negligible cytotoxicity and good cytocompatibility.

The antibacterial performance of the threads was further evaluated against *S. aureus* and *E. coli*. As shown in Figure 4e, AB-CCNF/SA threads exhibited clear inhibition zone with diameters of approximately 33 mm for *S. aureus* and 17 mm for *E. coli*, indicating strong antibacterial activity resulting from the release of TH. The inhibition zone against *S. aureus* was larger than that against *E. coli*, which can be attributed to the simpler cell wall structure of Gram-positive bacteria [55]. In contrast, neither collagen threads nor pristine CCNF threads exhibited antibacterial activity against either bacterial strain.

Overall, the above in vitro experiment results demonstrate that AB-CCNF/SA threads exhibit degradation behavior comparable to that of clinical collagen threads, while simultaneously providing improved thermal stability, excellent cytocompatibility, and effective antibacterial activity. These properties highlight the potential of AB-CCNF/SA threads as promising absorbable materials for minimally invasive facial thread lifting applications.

### 3.5. Model In Vivo Application Performance

**Figure 5:**
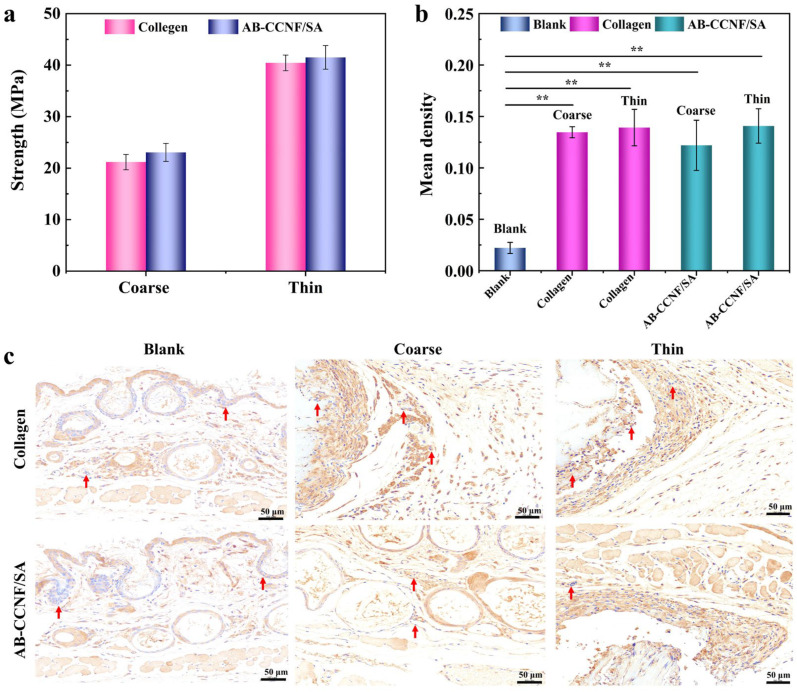
Strength and histological analysis of thread samples. (**a**) Tensile strength of AB-CCNF/SA and collagen threads with different diameters (0.45–0.50 mm vs. 0.15–0.20 mm), (**b**) Mean integral optical density of IL-6 immunostaining, (**c**) IL-6 immunostaining images of granulation tissue in Blank, Collagen and AB-CCNF/SA groups, the red arrow points to the phenomenon of inflammatory cell infiltration, ** *p* < 0.01, *n* = 5, scale bar represents 50 μm.

Different facial regions required lifting threads with distinct mechanical properties and diameters. In clinical practice, thicker threads are typically used in areas requiring stronger lifting support, such as severe facial sagging and neck wrinkles, whereas thin threads are commonly used in more delicate regions such as the outer nasolabial folds and the middle layer of the malar fat pad [56,57]. Therefore, two types of AB-CCNF/SA threads with different diameters were prepared in this study: coarse threads (0.45–0.50 mm) and thin threads (0.15–0.20 mm). Commercial collagen threads with the same specifications were used as controls. Tensile testing demonstrated that AB-CCNF/SA threads exhibited higher mechanical strength than collagen threads across both diameter ranges (Figure 5a). This improved structural integrity provides sufficient structural support during the lifting process and helps maintain the lifting effect over time.

To evaluate the in vivo tissue response, AB-CCNF/SA and collagen threads were implanted subcutaneously and harvested after 21 days for histological analysis. Quantitative immunohistochemical analysis of interleukin-6 (IL-6), a key inflammatory marker, was performed in the surrounding tissues. As shown in Figure 5b, the mean integrated optical density (IOD) of IL-6 staining was significantly higher in both thread-implanted groups compared with the blank control (*p* < 0.01). Histological observations (Figure 5c) further confirmed that both collagen and AB-CCNF/SA threads induced inflammatory cell infiltration in the surrounding subcutaneous tissue, which is characteristic of a typical foreign body response. Importantly, no statistically significant difference in inflammatory severity was observed between the AB-CCNF/SA and collagen groups. These findings indicate that AB-CCNF/SA threads exhibit in vivo biocompatibility comparable to that of clinically used collagen threads, while providing improved mechanical properties and intrinsic antibacterial functionality.

**Figure 6:**
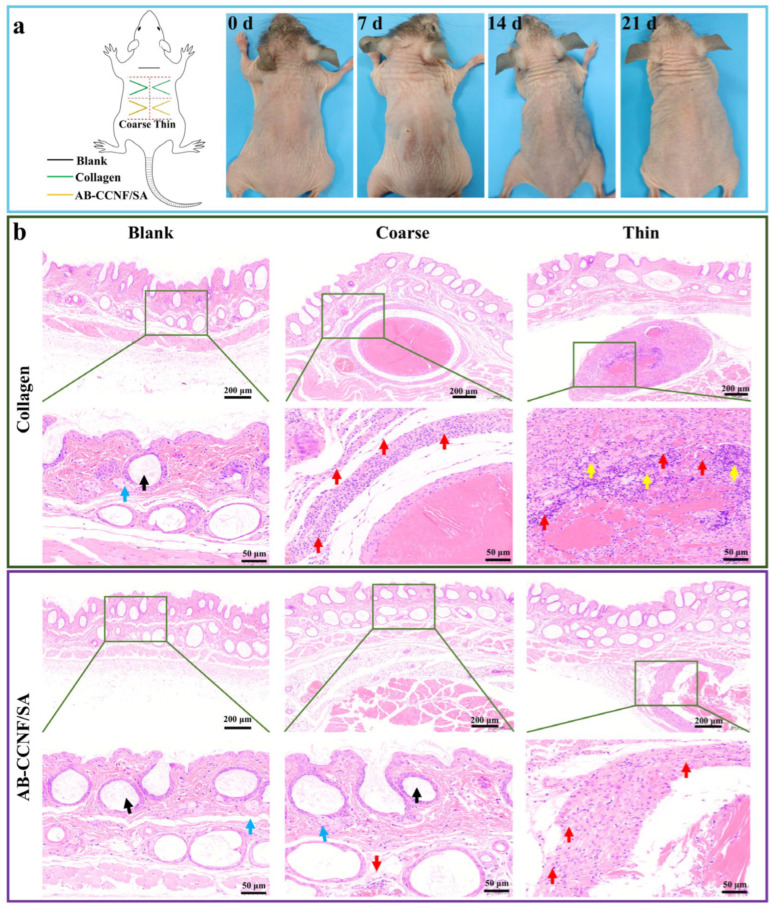
Performance evaluation of thread samples in skin tissue remodeling of mice and the corresponding histological analysis. (**a**) Digital image of absorption process after thread implantation, (**b**) H&E staining of skin tissues (the black arrows indicate hair follicles, the blue arrows indicate sebaceous glands, the red arrows indicate inflammatory cell infiltration, and the yellow arrows indicate mild nuclear condensation).

The influence of AB-CCNF/SA threads on skin tissue remodeling was further evaluated by subcutaneous implantation in nude mice. Macroscopic observations revealed that implantation of both coarse and thin threads resulted in visible improvement of dorsal skin folds compared with the untreated control (Figure 6a), and the lifting effect was comparable to that of collagen threads. In addition, implantation sites of thin threads exhibited nearly scarless healing within 4 days, whereas implantation sites of coarse threads required slightly longer recovery periods.

Histological analysis by H&E staining revealed different tissue responses among the groups (Figure 6b). In the control group (needle puncture only), the skin structure remained intact, showing normal epidermis, well-organized dermal collagen fibers, and abundant hair follicles (black arrows) and sebaceous glands (blue arrows) without obvious inflammatory infiltration. In contrast, the collagen thread group exhibited moderate inflammatory cell infiltration (red arrows) surrounding the implanted materials which is consistent with a typical foreign body response [58]. Interestingly, the AB-CCNF/SA thread groups showed favorable tissue responses. The thin AB-CCNF/SA thread group showed localized cellular responses such as mild nuclear condensation (yellow arrows), while the coarse AB-CCNF/SA thread group exhibited improved tissue integration characterized by relatively loose collagen organization, infiltration of hair follicle and sebaceous glands, and reduced inflammatory cell density compared with both the control and collagen groups.

Overall, these findings suggest that AB-CCNF/SA threads provide good in vivo biocompatibility and tissue integration, while maintaining sufficient mechanical support with high potential in cosmetic lifting applications.

## 4. Conclusions

This study presents a multifunctional AB-CCNF/SA thread fabricated via wet spinning strategy by integrating a CCNF/SA matrix with a drug-loaded ZIF-8 antibacterial delivery system. Notably, the CCNF was prepared from the solid residue of the traditional medicinal plant *Astragalus membranaceus* after extraction of active substances, offering a new utilization passway of residue of medicinal plant. Although the obtained *Astragalus* cellulose fibers contained a small amount of hemicellulose (4.51%) and lignin (6.71%), the presence of these impurities did not affect the preparation of CCNF and the performance of the resultant AB-CCNF/SA thread. The wet spinning conditions were optimized based on the mechanical performance of the resulting threads, yielding the following optimal parameters: CCNF/SA mass ratio of 1:1, total solid content of 1.3%, ZIF-8 loading of 6 wt.%, a coagulation bath containing 5% CaCl_2_, and post-spinning crosslinking in 2% glutaraldehyde for 1 h. Owing to the microporous structure of ZIF-8, the optimized threads exhibited a high drug loading capacity and encapsulation efficiency (19.3% and 97.1%, respectively). In addition, sustained drug release was achieved over 20 h without an obvious burst release, indicating the potential for prolonged antibacterial protection. In contrast, conventional collagen threads lack intrinsic antimicrobial properties. The AB-CCNF/SA threads also demonstrated favorable degradability, good cytocompatibility, and improved thermal stability. Furthermore, in vivo subcutaneous implantation in nude mice revealed inflammatory responses comparable to those of collagen threads and no severe foreign body reactions, confirming the good biocompatibility of the AB-CCNF/SA threads. Overall, the developed AB-CCNF/SA threads integrate a sustainable cellulose matrix (derived from medicinal plant residue), mechanical lifting support, sustained antimicrobial drug release, and biodegradable structural functions. These results suggest that the AB-CCNF/SA threads represent a promising candidate for next-generation absorbable threads in minimally invasive aesthetic and biomedical applications.

## Data Availability

The raw data supporting the conclusions of this article will be made available by the authors on request.

## Supplementary Materials

The following supporting information can be downloaded at: https://www.mdpi.com/article/10.3390/polym18101186/s1, Figure S1: Carboxyl content of CCNF sample; Figure S2: Viscosity changes of CCNF and CCNF/SA suspension; Figure S3: Strength of threads with different drug loadings; Figure S4: Strength of threads with different dosages of crosslinking agent; Figure S5: Standard curve of TH in methanol; Figure S6: (a) Standard curve of TH in PBS, (b)Standard curve of TH in saline, (c) Zero-order model, (d) First-order model, (e) Higuchi model, (f) Korsmeyer-Peppas model; Table S1: Data of kinetic and mathematical models for drug release mechanism.

## Abbreviations

The following abbreviations are used in this manuscript:

CCNF	carboxymethylated cellulose nanofibrils
SA	sodium alginate
ZIF-8	zeolitic Imidazolate Framework-8
TH	tetracycline Hydrochloride
*S. aureus*	*S**taphylococcus aureus*
*E. coli*	*Escherichia coli*
