# Supplementary material for: MOF-Enabled Nanocellulose Composite Threads for Sustained Antibacterial Drug Delivery and Minimally Invasive Soft-Tissue Lifting
Source: Polymers (Basel). 2026 May 12;18(10):1186. doi: 10.3390/polym18101186 (PMC13210424; doi:10.3390/polym18101186)
Supplement: Supplementary file 1 [file polymers-18-01186-s001.zip › polymers-4299974-supplementary.pdf]

polymers

MDPI

Article

MOF-Enabled Nanocellulose Composite Threads for Sustained Antibacterial Drug Delivery and Minimally Invasive Soft-Tissue Lifting

Meng Sun [1,2,†], Meiyan Wu [2,†], Ping Wang [2], Bing Li [3], Guang Yu [2], Haishun Du [4], Tao Lou [1,*] and Bin Li [2,*]

[1] College of Chemistry and Chemical Engineering, Qingdao University, Qingdao 266071, China; s13127235826@163.com
[2] State Key Laboratory of Photoelectric Conversion and Utilization of Solar Energy, Qingdao New Energy Shandong Laboratory, System Integration Engineering Center, Qingdao Institute of Bioenergy and Bioprocess Technology, Chinese Academy of Sciences, Qingdao 266101, China; wumy@qibebt.ac.cn (M.W.); wangping@qibebt.ac.cn (P.W.); yuguang@qibebt.ac.cn (G.Y.)
[3] Qingdao Hospital of Traditional Chinese Medicine (Municipal Hiser Hospital), Qingdao 266033, China; libing@qdu.edu.cn
[4] Department of Forestry, Michigan State University, East Lansing, MI 48824, USA; hdu@msu.edu
* Correspondence: taolou72@aliyun.com (T.L.); libin@qibebt.ac.cn (B.L.)
† These authors contributed equally to this work.

1. Experimental section

1.1. Preparation of CCNF

In this work, CCNF was prepared using *Astragalus* fiber as the fiber source material. *Astragalus membranaceus* (AM) as a traditional Chinese medicine is widely planted in Southeast Asia, Europe, and North America [1]. After extraction of active substances in pharmaceutical factory, the AM residue as solid waste has not been well utilized, causing the waste of resources. Herein, AM root (batch number 240503) was obtained from Qingdao Tiancheng Chinese Herbal Medicine Co., Ltd., China. The extraction of active substances was conducted according to a standard approach with slight modifications [1]. Briefly, 100 g AM root was boiled at 100 °C together with 10 L deionized water for 30 min, and then followed by solid-liquid separation. The obtained AM solid was further boiled with 10 L fresh deionized water through the same procedure. After that, the obtained AM residue was air-dried and then further purified by sulfonation treatment with 20 wt.% Na_2SO_3 (based on the dry mass of AM residue) and solid to liquid ratio of 1:8 at 140 °C for 2 h. Upon completion of sulfonation treatment, the *Astragalus* fiber was separated by filtration (through a Nylon bag with meshes 300), thoroughly washed with deionized water until neutrality, and then oven dried at 60 °C overnight.

The obtained *Astragalus* fiber (with 83.81% of cellulose, 4.51% of hemicellulose, and 6.71% of lignin, as determined following NREL procedure (NREL/TP-510-42618, 2011)) was functionalized via carboxymethylation according to a reported method with slight modifications [2]. Detailly, the air-dried astragalus fiber (30 g) was first swollen in ethanol (300 mL) for 5 min and then drained. Alkalization and etherification agents were prepared by dissolving NaOH (21.6 g) in an ethanol/water mixture (225/90 mL, v/v) and chloroacetic acid (21.6 g) in ethanol (585 mL), respectively. The fiber suspension and the chloroacetic acid solution were mixed in a reaction vessel and heated to 70 °C, after which the NaOH solution was added. The reaction mixture was maintained at 90 °C for 1 h under continuous stirring. Upon completion of the reaction, the carboxymethylated fibers were thoroughly washed with ethanol and then repeatedly washed with deionized water to remove residual chemicals. The resulting carboxymethylated fiber dispersion was subsequently

homogenized using a high-pressure homogenizer at 700 bar for 30 min to obtain the carboxymethylated cellulose nanofibrils (CCNF) dispersion.

1.2. Calculate the carboxyl content of CCNF

The carboxyl content of CCNF was determined by conductivity titration according to a reported method [3]. In detail, a small amount of CCNF (0.3 g, oven dried) was dispersed in deionized water (55 mL), followed by the addition of 0.01 mol/L NaCl (5 mL). The dispersion was acidified to pH 2.5-3.0 using 0.1 mol/L HCl and subsequently titrated with 0.1 mol/L NaOH under continuous magnetic stirring. The NaOH solution was added in 100 μL increments at 1 min intervals while the conductivity was continuously recorded. The carboxyl content was calculated using the following equation:

$$\textit{Carboxyl content}\ (\text{mmol/g}) = (CV_2 - CV_1) \times /m \times 100 \quad (1)$$

Where, C is the concentration of the standard NaOH solution (mol/L), V_1 is the volume of standard NaOH solution consumed in the early stable stage of the curve (mL), V_2 is the volume of standard NaOH solution consumed in the later stable stage of the curve (mL), and m is the oven-dry mass of CCNF (g).

1.3. Preparation of ZIF-8 and TH-loaded ZIF-8

ZIF-8 nanocrystals were prepared at room temperature in methanol following a previously reported procedure [4]. Certain amounts of 2-MeIm (1.2978 g) and $Zn(NO_3)_2{\cdot}6H_2O$ (0.5866 g) were dissolved in methanol (30 mL), respectively, with magnetic stirring (500 rpm, 15 min) and then sonication (200 W, 15 min). The zinc precursor ($Zn(NO_3)_2$) solution was subsequently added to the 2-MeIm solution and then stirred for 2 h. After reaction, the resulting white precipitate (i.e. ZIF-8 nanocrystals) was isolated via centrifugation (6440 *g*, 5 min), rinsed thrice with methanol, and then oven dried at 100 °C for 12 h.

TH-loaded ZIF-8 was prepared by the impregnation method. ZIF-8 (2 mg/mL) and 2 mL tetracycline hydrochloride (TH) (5 mg/mL) were simultaneously dispersed in anhydrous methanol with magnetic stirring, and reacted at room temperature for different time (1 h, 2 h, 4 h, 6 h, 8 h, 12 h, 24 h) accordingly. After that, the precipitate was collected and washed with anhydrous methanol to remove the unencapsulated TH. Finally, the TH-loaded ZIF-8 was obtained by oven drying (60 ℃, 20 min).

Drug encapsulation efficiency (DEE) and drug loading rate (DLR) were evaluated as the key metrics of carrier performance [5]. As mentioned, ZIF-8 (4 mg) was incubated with TH (5 mg/mL, 2 mL) in methanol under stirring at room temperature for predetermined time intervals. After incubation, the mixture was centrifuged (6440 *g*, 5 min), and then the obtained precipitate was washed thoroughly with anhydrous methanol to yield drug-loaded ZIF-8, while the supernatant derived from the first centrifugation was analyzed by UV-vis spectrophotometry at 360 nm to determine free TH concentration. DEE and DLR were calculated using Equations (2) and (3), respectively, with triplicate measurements for each sample.

$$DEE = W_{\text{drug-loaded}} / W_{\text{drug-added}} \times 100\% \quad (2)$$

$$DLR = W_{\text{drug-loaded}} / (W_{\text{drug-added}} + W_{\text{ZIF-8}}) \times 100\% \quad (3)$$

Where, $W_{drug\text{-}added}$ refers to the weight of TH added to the culture medium, $W_{drug\text{-}loaded}$ indicates the weight of TH loaded on ZIF-8, and $W_{ZIF\text{-}8}$ is the weight of ZIF-8.

1.4. Drug release model

The measured drug release data were fitted using the commonly used mathematical models, such as the Zero-order kinetic model (4), First-order kinetic model (5), Higuchi model square root law (6), and Korsmeyer-Peppas (K-P) model (7) to study the release

mechanism of TH. The following is a brief overview of the four common drug release kinetic models: the linearized forms of these models are presented below:

$$ln^{(M_t/M_0)} = ln_{k0} + ln_t \quad (4)$$

$$ln^{(1-M_t/M_\infty)} = -k_1 t \quad (5)$$

$$\frac{M_t}{M_\infty} = k_2 t^{\frac{1}{2}} \quad (6)$$

$$\frac{M_t}{M_\infty} = k_3 t^n \quad (7)$$

Where, M_t/M_∞ represents the cumulative release percentage of TH at time t; k_0, k_1, k_2, and k_3 are the corresponding constants for each model, and n is the release exponent. When $n \leq 0.45$, the release mechanism was mainly controlled by Fickian diffusion. When $n > 0.89$, the release mechanism was dominated by the second-type transport mechanism. However, if the value of n is between 0.45 and 0.89, it corresponds to a non-Fickian diffusion mechanism [6].

1.5. In vitro experiments

The cell viability of AB-CCNF/SA threads was measured to evaluate the cytotoxicity on human umbilical vein endothelial cells (HUVECS). HUVECS were first seeded into 96-well plates at a density of 5,000 cells/well and cultured in 200 μL of Dulbecco's Modified Eagle Medium (DMEM) containing 10% fetal bovine serum (FBS) for 24 h. The threads were then cut into small pieces, ground, and sterilized. Different doses of threads (0.15 mg/mL, 0.25 mg/mL in culture medium) were added to the wells and incubated at 37 °C for 24 h. After removing the thread samples, fresh DMEM (200 μL) was added to replace the old medium, and 20 μL of 3-(4,5-dimethylthiazol-2-yl)-2,5-diphenyltetrazolium bromide (MTT) solution was added, followed by incubation for another 4 h. After removing the medium again, 150 μL of dimethyl sulfoxide (DMSO) solvent was added. Absorbance was measured at 450 nm using a microplate reader (SMR60047, USCNK), and each sample was tested in triplicate. The cell viability of different samples was assessed using FBS-containing medium as the control [7]. After 24 h of culture, HUVECS were washed for 30 min with a Calcein/PI mixed solution [8]. The cytotoxicity of different thread was evaluated using an inverted fluorescence microscope (MI52-I, Guangzhou Mingmei Optoelectronics Technology Co., Ltd.).

$$Cell\ viability\ (\%) = \frac{A_s - A_c}{A_b - A_c} \times 100\% \quad (8)$$

Where, A_s is the absorbance of the samples, A_b is the absorbance of the blank sample, and A_c is the absorbance of the control sample.

The *in vitro* antibacterial experiments against *Escherichia coli* (*E. coli*, DH5-Alpha) and *Staphylococcus aureus* (*S. aureus*, ATCC29213) were performed as follows [9]: The antibacterial performance was evaluated using the agar plate diffusion method, which was assessed by the size of the inhibition zones formed. *E. coli* and *S. aureus* were cultured in an incubator at 37 °C for 24 h. After good growth, the cultures were diluted 100 times with medium for later use. Using a pipette, 100 μL of the bacterial solution was taken and evenly spread onto agar plates with a sterilized and cooled triangular glass rod. Sterilized tweezers were used to pick up the threads after cooling, and they were placed onto the agar medium that had been evenly coated with the bacterial solution. The culture plates were then incubated upside down in a 37 °C incubator for 24 h. The diameter of the inhibition zones was measured using a caliper at three different positions, and the average value was considered as the width of the inhibition zone.

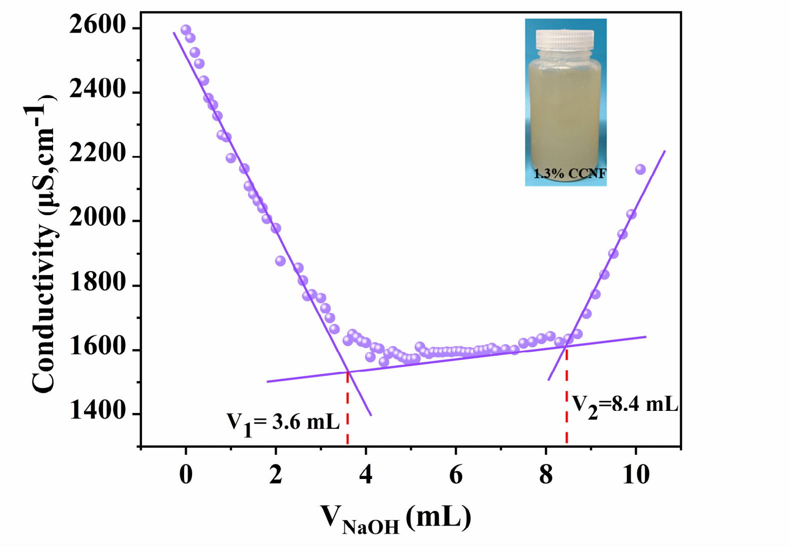

Figure. S1. Carboxyl content of CCNF sample.

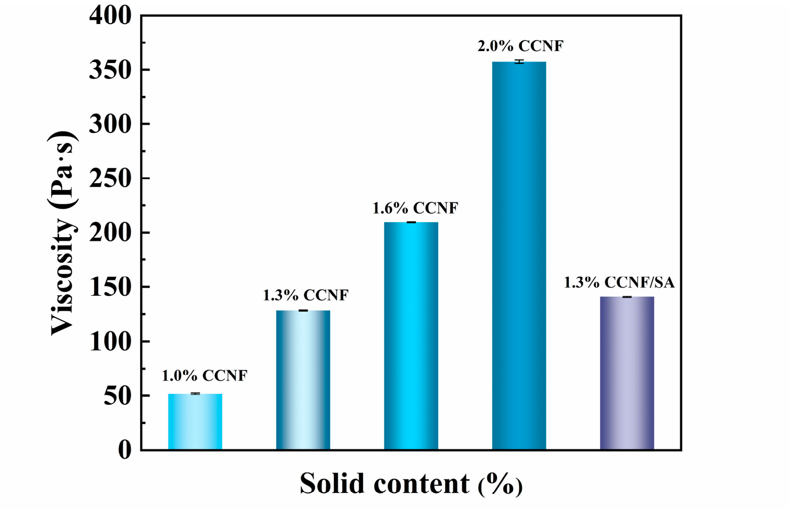

Figure. S2. Viscosity changes of CCNF and CCNF/SA suspension.

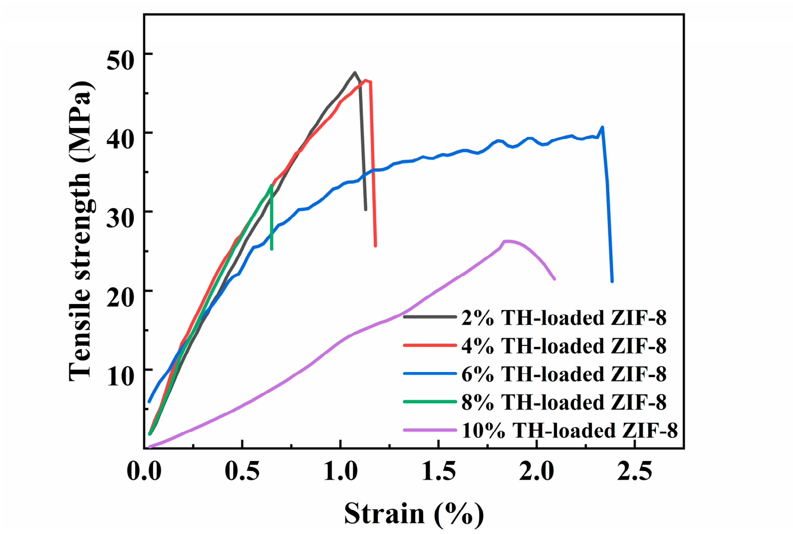

Figure. S3. Strength of threads with different drug loadings.

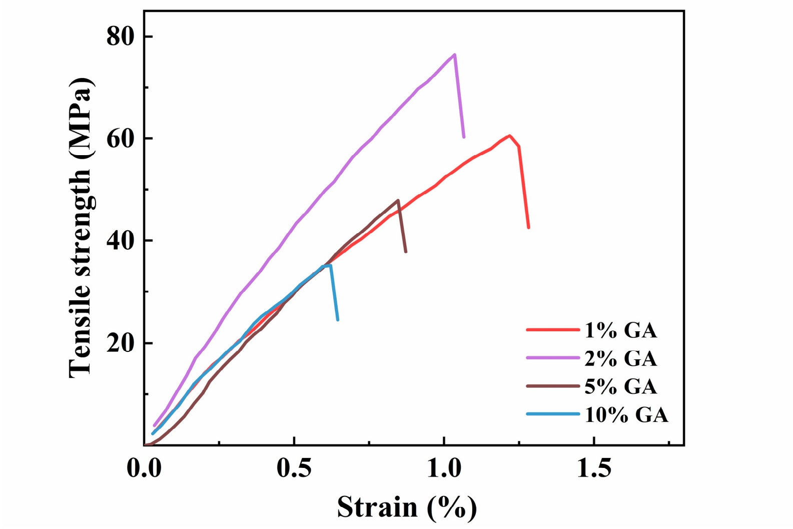

Figure. S4. Strength of threads with different dosages of crosslinking agent.

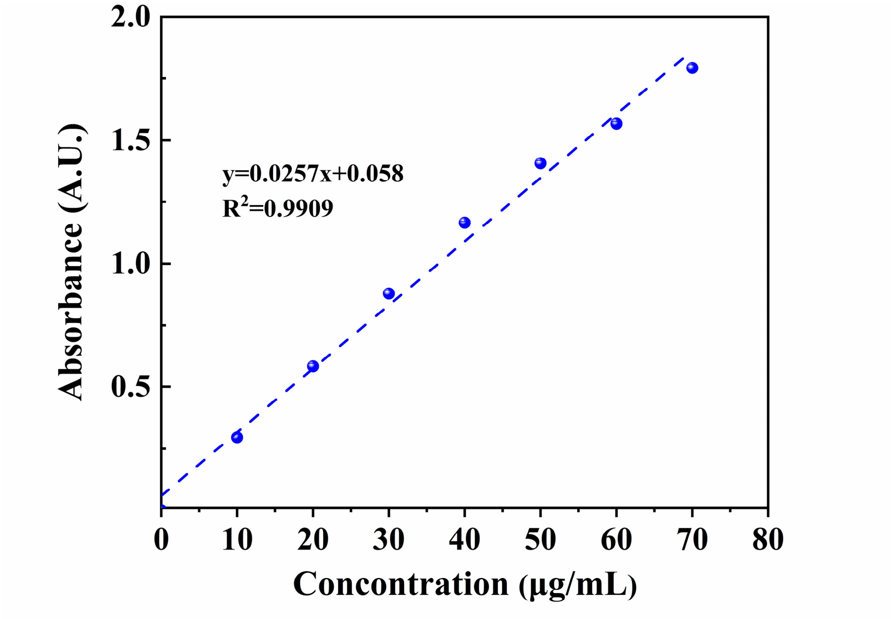

Figure. S5. Standard curve of TH in methanol.

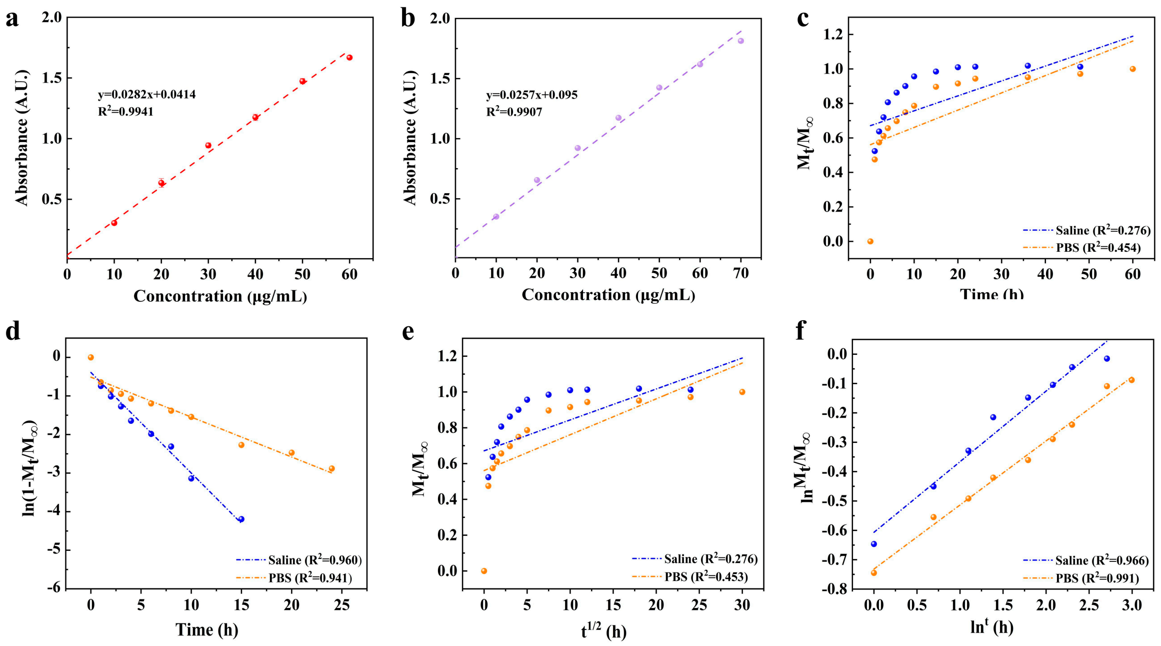

Figure. S6. (a) Standard curve of TH in PBS; (b)Standard curve of TH in saline; (c) Zero-order model; (d) First-order model; (e) Higuchi model; (f) Korsmeyer-Peppas model.

Table S1 Data of kinetic and mathematical models for drug release mechanism

Solution	Zero order		First order		Higuchi		Korsmeyer-Peppas		
	K_0	R^2	K_1	R^2	K_H	R^2	K_0	n	R^2
PBS	0.010	0.454	0.104	0.941	0.020	0.453	0.490	0.218	0.991
Saline	0.009	0.276	0.261	0.960	0.017	0.276	0.545	0.240	0.966

References

1. Long, X.; Lu, Y.-L.; Guo, H.; Tang, Y.-P. Recent Advances in Solid Residues Resource Utilization in Traditional Chinese Medicine. *ChemistrySelect* **2023**, *8*, e202300383, doi:10.1002/slct.202300383.
2. Zhao, S.; Fang, Z.; Liu, Y.; Li, G.; Lin, X.; Chen, K.; Qiu, X. Enhancing Aging Resistance of Transparent Paper: Structural Modification of Wood Fibers via Carboxymethylation. *ACS Sustainable Chem. Eng.* **2024**, *12*, 11244–11252, doi:10.1021/acssuschemeng.4c02619.
3. Fraschini, C.; Chauve, G.; Bouchard, J. TEMPO-Mediated Surface Oxidation of Cellulose Nanocrystals (CNCs). *Cellulose* **2017**, *24*, 2775–2790, doi:10.1007/s10570-017-1319-5.
4. Zhang, S.; Yi, J.; Yuan, X.; Zhang, Z.; Shan, Z.; Wang, H. Fabrication and Characterization of Carrageenan-Based Multifunctional Films Integrated with Gallic acid@ZIF-8 for Beef Preservation. *International Journal of Biological Macromolecules* **2024**, *274*, 133319, doi:10.1016/j.ijbiomac.2024.133319.
5. Wang, S.; Wang, S.; Yang, L.; Wang, P.; Song, H.; Liu, H. pH-Responsive Aminated Mesoporous Silica Microspheres Modified with Soybean Hull Polysaccharides for Curcumin Encapsulation and Controlled Release. *Food Chemistry* **2024**, *454*, 139832, doi:10.1016/j.foodchem.2024.139832.
6. Abbasnezhad, N.; Zirak, N.; Shirinbayan, M.; Kouidri, S.; Salahinejad, E.; Tcharkhtchi, A.; Bakir, F. Controlled Release from Polyurethane Films: Drug Release Mechanisms. *Journal of Applied Polymer Science* **2020**, *138*, doi:10.1002/app.50083.
7. You, J.; Cao, J.; Zhao, Y.; Zhang, L.; Zhou, J.; Chen, Y. Improved Mechanical Properties and Sustained Release Behavior of Cationic Cellulose Nanocrystals Reinforeced Cationic Cellulose Injectable Hydrogels. *Biomacromolecules* **2016**, *17*, 2839–2848, doi:10.1021/acs.biomac.6b00646.
8. Kim, J.-H.; Kim, S.; So, J.-H.; Kim, K.; Koo, H.-J. Cytotoxicity of Gallium–Indium Liquid Metal in an Aqueous Environment. *ACS Appl. Mater. Interfaces* **2018**, *10*, 17448–17454, doi:10.1021/acsami.8b02320.
9. Liu, Y.; Fan, Q.; Huo, Y.; Li, M.; Liu, H.; Li, B. Construction of Nanocellulose-Based Composite Hydrogel with a Double Packing Structure as an Intelligent Drug Carrier. *Cellulose* **2021**, *28*, 6953–6966, doi:10.1007/s10570-021-03978-5.
